# Supplementary material for: Ketone Bodies Are Mildly Elevated in Subjects with Type 2 Diabetes Mellitus and Are Inversely Associated with Insulin Resistance as Measured by the Lipoprotein Insulin Resistance Index
Source: J Clin Med. 2020 Jan 23;9(2):321. doi: 10.3390/jcm9020321 (PMC7074331; doi:10.3390/jcm9020321)
Supplement: Supplementary file 1 [file jcm-09-00321-s001.pdf]

Supplementary Data

Title:

Ketone Bodies are Mildly Elevated in Subjects with Type 2 Diabetes Mellitus and are Inversely Associated with Insulin Resistance as Measured by the Lipoprotein Insulin Resistance Index

Supplementary Figure 1. Linearity of expected versus NMR-measured (A) β-hydroxybutyrate, (B) acetoacetate, and (C) acetone in serum.

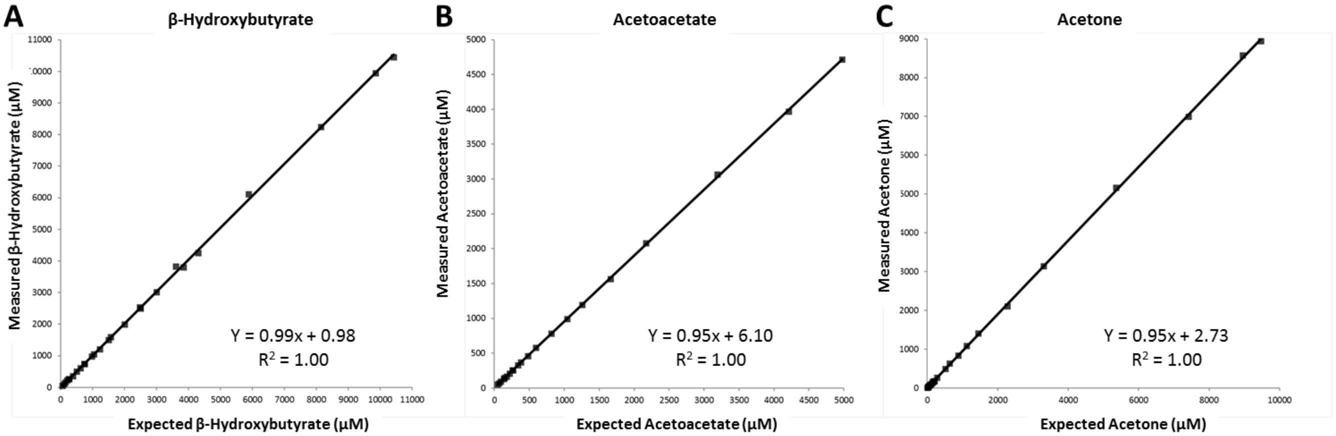

Supplementary Table 1. Linear regression results for tube comparison study; plain red-top serum, EDTA plasma tubes and sodium heparin plasma tubes compared to the Greiner LipoTube.

**Analyte**	**Plain Serum**				**EDTA Plasma**				**Heparin Plasma**			
	Slope	Intercept	R	% Bias	Slope	Intercept	R	% Bias	Slope	Intercept	R	%Bias
β-HB	1.00	-1.3	1.00	0.10	1.00	-2.0	1.00	-0.03	0.99	13.1	1.00	1.17
AcAc	1.02	-14.5	1.00	-4.19	1.00	-15.0	1.00	-6.01	1.03	-24.1	1.00	-7.27
Acetone	1.02	-1.8	1.00	1.70	1.01	-4.5	1.00	-0.09	1.01	-5.8	1.00	-1.16
Total KB	1.01	-19.0	1.00	-0.31	1.01	-22.2	1.00	-1.18	1.00	-15.9	1.00	-1.01

Abbreviations: AcAc, acetoacetate; β-HB, β-hydroxybutyrate; KB, ketone bodies.

Supplementary Table 2. Stability of ketone bodies at different storage conditions and multiple freeze-thaw cycles

Conditions	Hours/Days/Years /Cycles	N	Mean β-HB	% Bias β-HB	Mean AcAc	% Bias AcAc	Mean Acetone	% Bias Acetone
Room temperature	0 hour, baseline	3	437.0	--	291.9	--	267.9	--
	8 hours	3	423.4	-3.1	225.3	-22.8	250.0	-6.7
	24 hours	3	414.8	-5.1	156.9	-46.3	254.6	-5.0
	48 hours	3	411.2	-5.9	--	--	--	--
Refrigerated Temperature	0 hour, baseline	3	437.0	--	291.9	--	267.9	--
	24 hours	3	437.4	0.1	252.0	-13.7	248.3	-7.3
	48 hours	3	429.7	-1.7	233.0	-20.2	262.4	-2.1
	72 hours	3	449.8	2.9	--	--	--	--
Frozen (<-70 °C)	0 hour, baseline	3	437.0	--	291.9	--	267.9	--
	24 hours	3	427.2	-2.2	280.7	-3.8	226.9	-15.3
	48 hours	3	432.2	-1.1	277.5	-4.9	257.1	-4.0
	72 hours	3	445.4	1.9	294.5	0.9	270.5	1.0
	7 days	3	429.0	-1.8	283.3	-3.0	260.9	-2.6
	15 days	3	439.7	0.6	292.5	0.2	255.5	-4.6
Frozen (<-70 °C)	0 year, baseline	*	148.3	--	70.9	--	38.8	--
	3 years	*	148.3	0.0	50.9	-28.2	47.5	22.5
	6 years	*	146.1	-1.5	61.1	-13.8	46.2	19.1
Freeze-thaw cycles	0 hour, baseline	3	437.0	--	291.9	--	267.9	--
	1X	3	440.7	0.8	284.3	-2.6	241.5	-9.9
	2X	3	430.4	-1.5	295.6	1.2	273.5	2.1
	3X	3	440.8	0.9	287.9	-1.4	263.0	-1.8

Abbreviations: AcAc, acetoacetate; β-HB, β-hydroxybutyrate; *468 (β-HB), 214 (AcAc), 175 (Acetone).

Supplementary Table 3. Distribution of the analytes reported by the ketone body assay in 177,000 fasting and non-fasting subjects whose serum samples were tested in the clinical NMR laboratory.

	**Total Ketone Bodies (μM)**	**β-hydroxybutyrate (μM)**	**Acetoacetate (μM)**	**Acetone (μM)**
**0%**	<65.0	<45.0	<26.3	<19.7
**2.5%**	87.5	49.5	<26.3	<19.7
**25.0%**	149.7	97.2	28.0	<19.7
**50.0%**	201.2	134.2	39.7	28.5
**75.0%**	301.4	204.5	56.3	48.5
**97.5%**	987.1	693.9	143.4	179.5
**99.5%**	2,246	1,574	279.9	424.3
**100%**	20,331	10,152	2,655	8,503
